# Patient-reported outcomes on sleep quality and circadian rhythm during treatment with intravenous ketamine for treatment-resistant depression

**DOI:** 10.1177/20451253241231264

**Published:** 2024-03-04

**Authors:** Raymond Yan, Tyler Marshall, Atul Khullar, Travis Nagle, Jake Knowles, Mai Malkin, Brittany Chubbs, Jennifer Swainson

**Affiliations:** Department of Psychiatry, University of Alberta, Edmonton, Alberta, Canada; Department of Medicine, University of Calgary, Canada; Department of Psychiatry, University of Alberta, Edmonton, Alberta, Canada; Department of Psychiatry, University of Alberta, Edmonton, Alberta, Canada; Department of Psychiatry, University of Alberta, Edmonton, Alberta, Canada; Department of Psychiatry, University of Alberta, Edmonton, Alberta, Canada; Department of Psychiatry, University of Alberta, Edmonton, Alberta, Canada; Cabrini Center, 3rd Floor, 16811-88 Ave NW, Edmonton, AB, Canada T5R 5YR; Department of Psychiatry, University of Alberta, Canada; Misericordia Community Hospital, Edmonton, Alberta, Canada

**Keywords:** circadian rhythm, insomnia, ketamine, patient-reported outcomes, sleep quality, treatment-resistant depression

## Abstract

**Background:**

Intravenous (IV) ketamine is a rapid acting antidepressant used primarily for treatment-resistant depression (TRD). It has been suggested that IV ketamine’s rapid antidepressant effects may be partially mediated *via* improved sleep and changes to the circadian rhythm.

**Objectives:**

This study explores IV ketamine’s association with changes in patient-reported sleep quality and circadian rhythm in an adult population with TRD.

**Methods:**

Adult patients (18–64 years) with TRD scheduled for IV ketamine treatment were recruited to complete patient rated outcomes measures on sleep quality using the Pittsburgh Sleep Quality Index (PSQI) and circadian rhythm using the Morningness–Eveningness Questionnaire (MEQ). Over a 4-week course of eight ketamine infusions, reports were obtained at baseline (T0), prior to second treatment (T1), prior to fifth treatment (T2), and 1 week after eighth treatment (T3).

**Results:**

Forty participants with TRD (mean age = 42.8, 45% male) were enrolled. Twenty-nine (72.5%) had complete follow-up data. Paired *t* tests revealed statistically significant improvements at the end of treatment in sleep quality (PSQI) (*p* = 0.003) and depressive symptoms (Clinically Useful Depression Outcome Scale-Depression, *p* < 0.001) while circadian rhythm (MEQ) shifted earlier (*p* = 0.007). The PSQI subscale components of sleep duration (*p* = 0.008) and daytime dysfunction (*p* = 0.001) also improved. In an exploratory *post hoc* analysis, ketamine’s impact on sleep quality was more prominent in patients with mixed features, while its chronobiotic effect was prominent in those without mixed features.

**Conclusion:**

IV ketamine may improve sleep quality and advance circadian rhythm in individuals with TRD. Effects may differ in individuals with mixed features of depression as compared to those without. Since this was a small uncontrolled study, future research is warranted.

## Plain language summary

Patient-reported changes in sleep during treatment with intravenous ketamine for depression

Intravenous ketamine is a fast acting treatment for depression that does not respond to more conventional antidepressant medications. Almost all people with depression have problems with sleep as a symptom of their illness. This can include things like difficulties falling asleep, problems staying asleep, sleeping more or less than usual, and shifting the sleep schedule to stay up later than usual. It has been previously suggested that improving sleep in people with depression may be part of how ketamine exerts its antidepressant effect. This study surveyed patients with depression who received eight intravenous infusions of ketamine (two per week for 4 weeks) to ask them about their sleep quality and patterns before treatment, part way through their course of treatment and after the treatments were completed. Symptoms of depression were also measured. Data were collected on 29 people. People reported overall that sleep quality did improve with ketamine treatments, and that timing of sleep shifted earlier. Sleep duration increased and people had less problems with daytime functioning. There is a subtype of depression called depression with “mixed features,” meaning that these people, in addition to being depressed, may have some activating symptoms like irritability, restlessness, and agitation. It is thought that this type of depression may be biologically different from depression without these symptoms. In this study, around half (15/29) had mixed features. Sleep quality improved only in the group without mixed features. On the other hand, the group with mixed features had their sleep schedule shift earlier, but the group without mixed features did not. This supports the idea that these two types of depression may be biologically different, and ketamine treatment may exert different effects on the sleep of each group. This was a small study, but suggests a need for future research.

## Introduction

Depressive disorders are considered the leading cause of disability worldwide, affecting approximately 4% of the global population.^1^ With existing conventional antidepressants, only around 30% of patients experience full recovery, while the rest will either respond without remission or not at all.^2^ For patients with treatment-resistant depression (TRD), commonly defined as those failing at least two prior antidepressants of adequate dose and duration,^3^ there is an urgent need for novel treatments and strategies in reducing the symptoms and disease burden of the illness.

One promising approach gaining traction may be through the complex bidirectional link between depression and sleep.^4^ It is widely recognized that sleep is a core symptom in depressive disorders,^5^ with up to 90% of afflicted patients reporting disrupted sleep quality.^6^ Common subjective complaints include difficulties with falling asleep, staying asleep, early-morning awakening, daytime fatigue, or unrefreshing sleep.^7^ Sleep dysfunction, in turn, could precipitate depressive episodes, perpetuate affect dysregulation, worsen mood severity, or persist as a residual symptom.^8^ The presence of disrupted sleep is a risk factor for recurrence of depressive disorders.^9^ Neuro-psycho-biological studies implicate disturbances in sleep–wake homeostasis, biological clock functioning,^10^ inflammatory cytokines,^11^ neurocircuitry,^12^ synaptic-neuroplasticity,^13^ and altered neurotransmission as interacting key mechanisms that may explain an overlap in pathophysiology between sleep and depressive-like behaviors. Taken together, sleep is increasingly recognized to play a critical role in the maintenance of brain health, general well-being, and healthy cognition.^14^

Growing evidence is converging on recommendations to target comorbid sleep disturbances in depression.^15^ A recent meta-analysis suggested that treating insomnia may improve mood outcomes in patients with depression.^16^ This approach aligns with current recommendations from international experts to consider clinical features and patient comorbidities in personalizing pharmacotherapy and augmentation options.^17^ For instance, depressed patients with identified insomnia or circadian rhythm disturbances may be treated or augmented with agents that are considered sedating, or treatments that may resynchronize biological clock abnormalities.^18^ The latter strategy has evolved into its own burgeoning field – depressive symptoms have been associated with circadian-phase delay and eveningness,^19^ while phase advancement has been associated with improvement in depression.^20^ Studies demonstrating the effectiveness of chronotherapeutics such as light therapy, melatonin, cognitive behavioral therapy for insomnia, and sleep deprivation in treating depression with comorbid sleep disorders further emphasizes the importance of stabilizing sleep–wake cycles.^18^ Similarly, agomelatine, a drug that acts on the melatonin receptor, has been shown to help major depressive disorder (MDD) by stabilizing sleep wake cycles.^21^

Ketamine is a noncompetitive *N*-methyl-d-aspartate (NMDA)-antagonist, with a long history of use in pain medicine and anesthesia. More recently, intravenous (IV) ketamine given at subanesthetic doses has been established as a rapid acting antidepressant, with improvements in mood noted as early as 40 min postinfusion.^22^ A recent review citing clinical recommendations for IV ketamine use notes evidence of an antidepressant effect for up to 10 days postinfusion after a single dose of IV ketamine, that can extend to 18 days with repeated infusion.^23^ This is in stark contrast to the delayed effects with existing antidepressants, where at least 2 weeks of continuous treatment is required to achieve clinical improvement.^24^ Doses of 0.5–1.0 mg/kg IV ketamine have been recommended for treating depression.^23^

Ketamine is a noncompetitive antagonist at the NMDA receptor, thus modulating glutamate – it has been previously suggested that MDD is associated with dysregulated glutamatergic neurotransmission. However, the mechanism of action of ketamine’s antidepressant effect is thought to be much more complex, involving downstream pathways.^25^ Ketamine also interacts with the mechanistic target of rapamycin complex 1 pathway (mTORC1), which is associated with brain-derived neurotrophic factor (BDNF). BDNF is a growth factor protein involved in neurogenesis and synaptogenesis. As such, the mechanism of action involves a cascade of events where ketamine antagonizes NMDA receptors on γ-aminobutyric acid (GABA) interneurons, which in turn suppresses downstream glutamatergic neurons. This subsequently causes increased glutamate, which increases signaling in BDNF and mTORC1 pathways. Glutamate pathways in the retinothalamic tract can influence circadian rhythm, with ketamine surmised to modulate these pathways through the above mechanisms. One clinical study of patients with unipolar or bipolar depression previously reported that a single 0.5 mg/kg infusion of IV ketamine may correlate with advances in circadian rhythm and increased levels of 24 h motor activity, as measured by wrist actigraphy.^26^ Ketamine is also known to rapidly induce expression of clock genes,^27^ which are responsible for regulating circadian cycles. An emerging clinical application of interest suggests that ketamine may be used to treat the sleep disturbances and circadian rhythm disorders seen in MDD patients.^28^ Previously overlooked, research is now highlighting the importance of understanding ketamine’s impact on sleep as it may be critical in understanding its physiological effects.^29^

The clinical effects of IV ketamine on sleep in depressed individuals remain largely unreported. Three retrospective studies used patient-reported outcome measures (PROMs). A study of 323 patients with TRD received 4 IV ketamine infusions, starting at 0.5 mg/kg with the option to optimize to 0.75 mg/kg. Sleep outcomes were measured by sleep subitems on the self-rated Quick Inventory of Depressive symptoms Scale (QIDS-SR), with patients reporting improvements in insomnia, nighttime restlessness, and early-morning awakening. It was suggested that these sleep improvements may potentially mediate improvements in depressive symptoms, and predicted likelihood of response or remission.^30^ Another study of 123 patients with unipolar or bipolar depression receiving 6 IV ketamine infusions dosed at 0.5 mg/kg demonstrated improvements in sleep as measured by sleep subitems on the Hamilton Depression Scale (HAM-D). These improvements were associated with increases in BDNF, a known peripheral marker for neuroplasticity and thought to be important in ketamine’s synaptogenic effects.^31^ In contrast, a third report noted that sleep disturbances remained a common residual symptom of depression 2 weeks after a single 0.5-mg/kg infusion of IV ketamine.^32^

This preliminary data prompt the need for further research with validated, sleep-specific tools to further characterize ketamine’s impact on clinically relevant sleep outcomes. To date, one case study of a single patient included a sleep-specific validated tool (Pittsburgh Sleep Quality Index, PSQI) as a primary outcome with improvements in sleep quality observed with intranasal esketamine.^33^ A very recent report of 35 adult patients (age 60+) used only item 9 of the PSQI rather than the entire scale to assess sleep quality. They reported that 36% of patients reported fairly good or good sleep pre-ketamine, but with eight infusions dosed at 0.5 mg/kg, this number increased to 77%.^34^ There exists one study protocol for a prospective study using polysomnography to evaluate the effects of a course of IV ketamine on sleep and circadian rhythm, but results have not yet been published.^35^ The connection between ketamine and sleep remains understudied with lack of real-world data on the overall time course of antidepressant response to ketamine compared to sleep effects.^36^

To build on existing data in a real-world population, our study used the PSQI and the Morningness–Eveningness Questionnaire (MEQ), two validated sleep-specific PROMs. The PSQI evaluates patient perception of sleep quality while the MEQ reports on chronobiotic patterns. We tracked these self-reported sleep outcomes as well as mood ratings over the time course of eight IV ketamine infusions. If IV ketamine was effective for treating sleep disturbances with depression, we hypothesized that the overall sleep quality would improve over the course of treatment, while the circadian rhythm would advance with patients beginning to sleep earlier in the night, as per effects of other chronotherapeutic interventions with antidepressant effects.^18^

## Methods

### Design and clinical setting

Data were collected from January 2020 to January 2022 from the IV ketamine programs at two community hospitals (Misericordia Community Hospital and Grey Nuns Hospital) in Edmonton, Alberta, Canada. Patients receiving IV ketamine for TRD (defined as a failure to respond to at least two prior antidepressants) were recruited to participate by completion of PROMs at four time points throughout their treatment; prior to first treatment (T0), prior to second treatment (T1), prior to fifth treatment (T2), and 1 week after the eighth and final treatment (T3). PROMs used in the study included the Clinically Useful Depression Outcome Scale-Depression (CUDOS) and the Clinically Useful Depression Outcome Scale-Mania (CUDOS-M), as well as the PSQI and MEQ. The study was approved by University of Alberta Research Ethics Board (REB ID: PRO00094438). The reporting of this study conforms to the Strengthening the Reporting of Observational Studies statement.^37^

### Participant eligibility criteria

Patients (18–64 years) who were scheduled to undergo IV ketamine treatment for TRD, defined as a failure to respond to two or more antidepressant trials, were eligible to participate in our study. The clinical treatment protocol at both hospital sites excluded patients from ketamine treatment who had current or active psychotic symptomatology, poorly controlled hypertension, unstable medical conditions (e.g. cardiovascular and respiratory disease), active substance use disorders, pregnant/breastfeeding, or any prior documented intolerance/allergies to IV ketamine. These exclusion criteria were interpreted at the discretion of the treating psychiatrist. For our study, patients with bipolar depression were excluded, as were any patients previously been treated with IV ketamine.

### Informed consent

All enrolled participants provided written informed consent following review of study information including potential risks and benefits of study participation. Note that patients had previously consented to the course of IV ketamine treatment as part of the clinical care route with their treating psychiatrist.

### Exposure

IV ketamine treatment in the clinical protocol for both hospitals involved an infusion of 0.5–1.0 mg/kg over 40 min twice weekly for 4 weeks. This was administered according to the Alberta Health Services Ketamine for depression provincial protocol (Alberta Health Services, 2020). In this protocol, ketamine is initiated at a dose of 0.5 mg/kg and may be increased up to 1.0 mg/kg at the discretion of the treating physician. Treatments were administered between the hours of 08:00 h and 11:00 h, as per clinical programming.

### Data collection methods

Charts of all enrolled patients were reviewed for demographic information and medical history. PROMs were collected by a member of the study team at each of the four data collection time points.

## Primary outcome

### Sleep quality

The PSQI is a validated PROM of sleep quality and sleep disturbances, and was used to assess self-reported sleep outcomes over the course of IV ketamine treatment. Its seven subscale components include subjective sleep quality, sleep latency, sleep duration, habitual sleep efficiency, sleep disturbances, use of sleeping medication, and daytime dysfunction. Global PSQI scores range from 0 to 21, with a total score greater than 5 validated to have diagnostic sensitivity of 89.6% and specificity of 86.5% in distinguishing between good and poor sleepers.^38,39^

## Exploratory outcomes

### Circadian rhythm

The MEQ is a widely used PROM for evaluating circadian rhythm and sleep rhythm patterns.^40^ Individuals scoring higher than 58 and lower than 42 are classified as morningness-type and eveningness-type, respectively.^41^ Depressive symptoms are thought to be associated with circadian-phase delay and eveningness. Changes in circadian rhythm can be monitored over treatment courses.^42^

### Symptoms of depression

The CUDOS is a validated PROM^43^ that closely correlates with the Hamilton Depression (HAM-D) scale,^44^ which, though not always used due to time constraints associated with clinician rating, is considered the gold standard in controlled studies.^45^ The CUDOS is divided into three subscales; the depressive (CUDOS-D) subscale,^46^ a mixed features subscale (CUDOS-M), and a functional impairment subscale. Clinical response is defined as a 50% or more improvement in scores,^43^ while scores of 0–10 have been noted as a valid indicator of remission.^47^

### Presence of mixed mood symptoms

Patients with unipolar depression are not a homogeneous population, and the presence of mixed features is increasingly becoming recognized as a unique subpopulation of depressed individuals.^48^ As these individuals may exhibit differing sleep profiles, patients were screened for mixed features using the CUDOS-M, developed as a reliable and valid measure of the DSM-5 mixed features specifier for MDD.^49^ On this scale, patients were asked about manic symptoms occurring during their depression including (1) feeling happy and cheerful like a high, (2) experience of brilliant and creative ideas, (3) feelings of extreme self-confidence, (4) sleeping only a few hours but waking with lots of energy, (5) endless energy level, (6) more talkative than usual, (7) taking faster than usual, (8) racing thoughts, (9) taking on new projects with a feeling of being able to do anything, (10) being more social and outgoing that usual, (11) doing wild, impulsive things, spending more freely than usual, and, (12) thoughts and fantasies about sex more than usual, and (13) felt so happy and cheerful it was like a high. Each item was scored from 0 to 4, and a total score of 7 or greater indicates the presence of mixed features (sensitivity 0.96; specificity 0.71).^50^

### Study size

*A priori*, a power of 0.80 and desired effect size of 0.6 was used for a total sample size of 32 participants was recommended at study design stage. However, during the data collection stage, given the percent attrition rate, the team decided to increase the total sample size to 40 with aims to account for missing follow-up data.

### Statistical analysis

Patient baseline demographic characteristics were reported narratively and in a summary table. Any participants who did not complete the course of eight discontinued IV ketamine treatments for any reason prior to the completion of eight treatments were considered lost to follow up and not included in the primary outcome. However, they were included in longitudinal data sets to monitor for any changes over time.

Consistent with the research objectives, a descriptive analysis consisting of a series of pre–post tests was conducted on each outcome variable. Continuous outcome variables were first compared using a two-tailed Pearson *R* bivariate correlation matrix to examine for multicollinearity using outcomes from baseline. Variables with an *R* value of greater than ±0.75 (*p* < 0.01) were consolidated. Each outcome variable was then examined for normal distribution. Tests of the two *a priori* hypotheses were conducted using alpha levels of 0.05 per two-tailed test.

Individuals with missing data at T0 or T3 were removed from the paired *t* test analysis. *Post hoc* analyses were conducted and explored descriptively and qualitatively. IBM SPSS Statistics for Windows (Version 25.0; IBM Corp., Armonk, NY, USA) was used to carry out the statistical analyses.

## Results

### Patient clinical and demographic characteristics

**Figure 1.**
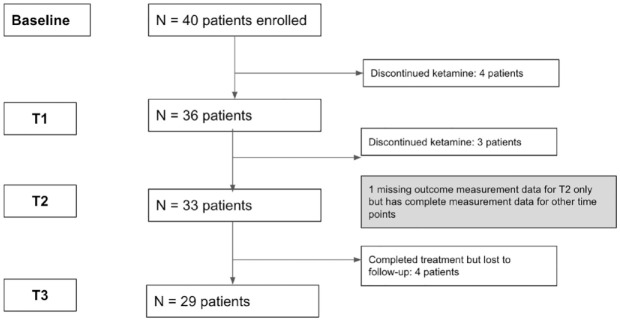
Study flowchart.

Forty participants with TRD were enrolled into the study and provided baseline data at T0. Twenty-nine participants (72.5%) completed all eight ketamine treatments and provided T3 data at the 1 week follow-up. Of the 11 individuals with incomplete data sets, 4 individuals who completed the course of ketamine treatments did not follow up to complete the T3 questionnaires 1 week later. Seven enrolled participants (17.5%) did not complete the full course of eight ketamine infusions, so were missing data at one or more time points. Figure 1 displays the study flowchart.

Participants ranged in age from 19 to 64 with a mean (SD) age of 42.8 (10.7) years. Forty-five percent were male. Nearly half of participants (19/41) endorsed mixed features with a CUDOS-M score greater than 7. Eighty percent of the participants reported five or more previous antidepressant trials, 60% reported previous electroconvulsive therapy (ECT), and 82.5% remained on a combination antidepressant therapy during treatment. The baseline mean (±SD, *n* = sample size) outcome values included PSQI = 12.31 (3.76, *N* = 40), MEQ = 47.8 (SD = 10.0, *N* = 40), CUDOS-D = 44.9 (SD = 6.61, *N* = 40), and QIDS-SR = 19.8 (SD = 3.29, *N* = 34). At baseline, 39 of the 40 (97.5%) patients were considered poor sleepers on the PSQI (Score > 5). See Table 1 for further information regarding baseline characteristics.

**Table 1.** Baseline characteristics of study population.

Characteristics	Value
Sample characteristics	
*N* enrolled at Baseline	40
*N* (%) with complete follow-up data	29 (72.5%)
Age years, mean (SD)	42.8 (10.7)
Gender, *n* (%) male	18 (45.0%)
Previous treatment history	
*N* lifetime antidepressant trials, *n* (%)	
<5 trials	8 (20.0%)
⩾5 trials	32 (80.0%)
Electroconvulsive therapy, *n* (%)	24 (60.0%)
Concurrent medications	
Antidepressants, *n* (%)	
Monotherapy	7 (17.5%)
Combination therapy	33 (82.5%)
Sleep medications, *n* (%)	
None	8 (20.0%)
Monotherapy	18 (45.0%)
Combination therapy	14 (35.0%)
Other concurrent psychiatric medications, *n* (%)	
Mood stabilizers	2 (5%)
Gabapentinoids	3 (7.5%)
Benzodiazepines	11 (27.5%)
Psychostimulants	6 (15%)
Antipsychotics	21 (52.5%)
Baseline sleep measures	
Sleep quality (PSQI Global Score), mean (SD)	12.31 (3.76)
Poor sleeper (PSQI > 5), *n* (%)	39 (97.5%)
Good sleeper (PSQI < 5), *n* (%)	1 (2.5%)
Baseline circadian rhythm	
Circadian rhythm (MEQ total score), mean (SD)	47.76 (10.01)
Eveningness-type (MEQ < 42), *n* (%)	10 (25%)
Intermediate-type (MEQ 42–58), *n* (%)	24 (60%)
Morningness-type (MEQ > 58), *n* (%)	6 (15%)
Baseline depression scores	
Depression (CUDOS-D score), mean (SD)	44.86 (6.61)
Depression (QIDS-SR score), mean (SD)	19.75 (3.29)
Mixed features on CUDOS-M	
Mixed feature scores greater or equal to 7, *n* (%)	19 (47.5%)
Mixed feature scores 6 or less, *n* (%)	21 (52.5%)

CUDOS-D, Clinically Useful Depression Outcome Scale-Depression; CUDOS-M, Clinically Useful Depression Outcome Scale-Mania; MEQ, Morningness–Eveningness Questionnaire; PSQI, Pittsburgh Sleep Quality Index; QIDS-SR, Quick Inventory of Depressive symptoms Scale; SD, standard deviation.

### Sleep and mood outcomes

All PROMs were compared at baseline (T0) and 1 week following the last ketamine infusion (T3). *T* test analyses were based on 29 completed assessments at T3 follow-up with *N* = 11 (27.5%) missing outcome data. Table 2 summarizes these results.

**Table 2.** Self-reported sleep and mood before (T0) and after (T3) a course of eight IV ketamine treatments.

Variable	T0 mean (SD)	T3 mean (SD)	Mean (SD) difference	*p*
PSQI Global Score	12.30 (3.76)	10.2 (4.10)	−2.07 (3.4)	0.003*
Component 1: subjective sleep quality	1.79 (0.77)	1.31 (0.71)	−0.48 (0.87)	0.006*
Component 2: sleep latency	2.24 (0.74)	1.93 (0.92)	−0.31 (0.89)	0.071
Component 3: sleep duration	1.00 (1.10)	0.52 (0.74)	−0.48 (0.91)	0.008*
Component 4: habitual sleep efficiency	1.34 (1.32)	1.21 (1.21)	−0.14 (0.88)	0.403
Component 5: sleep disturbances	1.65 (1.21)	1.59 (0.68)	−0.07 (0.59)	0.537
Component 6: use of sleep medications	2.17 (1.23)	2.14 (1.30)	−0.03 (0.78)	0.813
Component 7: daytime dysfunction	2.10 (0.62)	1.55 (0.87)	−0.55 (0.83)	0.001**
MEQ	47.8 (10.0)	50.0 (9.57)	+2.21 (4.10)	0.007*
CUDOS	44.86 (6.61)	26.28 (13.07)	−18.6 (12.5)	<0.001**

Bold font indicates a statistically significant change at the ⩾0.05 significance level. An asterisk (*) indicates significance at the ⩾0.01 level, ** indicates significance at the ⩾0.001 level.

Baseline (T0), prior to first ketamine treatment; CUDOS, Clinically useful Depression Outcome Scale; IV, intravenous; MEQ, Morningness–Eveningness Questionnaire; PSQI, Pittsburgh Sleep Quality Index; T3, 1 week after eighth ketamine treatment.

### Primary outcome: changes in patient-reported sleep quality

Participants reported a lower PSQI score at T3 than at baseline (Baseline mean = 12.30, SD = 3.76; T3 mean = 10.2, SD = 4.10; *p* = 0.003). Subitems that improved included subjective sleep quality, sleep duration, and daytime dysfunction.

### Exploratory outcome 1: circadian rhythm

On average, participants reported more delayed circadian rhythm at baseline (mean = 47.8, SD = 10.0) than at T3 (mean = 50.0, SD = 9.57). This improvement, 2.21 (4.63%), 99% CI [4.31–0.01] was statistically significant, *t*(−2.90) df = 28, *p* = 0.007. Four patients exhibited a change from baseline chronotype on the MEQ either from intermediate-type to a morning-type or from an evening-type to an intermediate-type.

### Exploratory outcome 2: depressive symptoms

Participants reported more depressive symptoms at baseline (mean = 44.9, SD = 6.61) than at T3 follow-up (mean = 26.3, SD = 13.1). This improvement, −18.6 (−41.4%), 99% CI [12.2–25.0] was statistically significant, *t*(7.99) df = 28, *p* < 0.001.

### Exploratory outcome 3: temporality of improvements with repeated IV ketamine on sleep, circadian rhythm, and depressive symptoms in patients with TRD

Depressive symptoms improved immediately from baseline to T1 (*p* < 0.001 on CUDOS-D), from T1 to T2 (*p* < 0.001), and then further from T2 to T3 (*p* < 0.001). Both sleep outcomes (PSQI and MEQ) did not significantly improve until T3 (*p* = 0.003 on PSQI, *p* = 0.007 on MEQ). See Table 3.

**Table 3.** Changes in sleep and depressive symptoms from baseline (T0) to follow-up (T1–T3).

Outcome	Instrument	Mean difference (*p* value)		
		T0–T1	T0–T2	T0–T3
Sleep – quality and disturbance	PSQI Global Score	0.17 (0.61)	**−0.91 (0.03)**	−2.07 (0.003)*
Sleep – circadian rhythm	MEQ	0.67 (0.26)	**1.47 (0.04)**	2.21 (0.007)*
Depressive symptoms	CUDOS-D	−6.17 (<0.001)**	−13.6 (<0.001)**	−18.6 (<0.001)**

Bold font indicates a statistically significant change at the ⩾0.05 significance level. An asterisk (*) indicates significance at the ⩾0.01 level, **indicates significance at the ⩾0.001 level.

CUDOS-D, Clinically Useful Depression Outcome Scale-Depression; MEQ, Morningness–Eveningness Questionnaire; PSQI, Pittsburgh Sleep Quality Index.

### Exploratory outcome 4: sleep quality and circadian rhythm in depressed patients with mixed features *versus* without mixed features

Of the 29 full data sets, 14 had a CUDOS-M score of 7 or greater suggesting presence of mixed features, while 15 had a CUDOS-M score of 6 or below suggesting the absence of mixed features.

In patients with mixed features, there was statistically significant improvement in sleep quality (PSQI scores, pre-post change of −3.64, *p* = 0.001) but no advancement in circadian rhythm (MEQ scores, pre-post change of 1.43, *p* = 0.21) from T0 to T3. Conversely, the participants without mixed features endorsed advancement in circadian rhythm (MEQ scores, pre-post change of 2.93, *p* = 0.016), but no improvement in sleep quality (PSQI scores, pre-post change of −0.6, *p* = 0.440) from T0 to T3 (Table 4).

**Table 4.** Sleep quality and circadian rhythm in depressed patients with mixed features *versus* those without mixed features.

Variable	*N* pairs	Pre mean (SD)	Post mean (SD)	Mean (SD) difference	*p*
Depression with mixed features					
PSQI Global Score	14	13.50 (3.03)	9.86 (3.37)	−3.64 (3.25)	0.001**
MEQ	14	51.21 (12.37)	52.64 (12.00)	1.43 (4.05)	0.210
Depression without mixed features					
PSQI Global Score	15	11.2 (4.13)	10.6 (4.78)	−0.60 (2.92)	0.440
MEQ	15	44.53 (5.95)	47.47 (5.96)	2.93 (4.15)	**0.016**

Bold font indicates a statistically significant change at the ⩾0.05 significance level. An asterisk (*) indicates significance at the ⩾0.01 level, **indicates significance at the ⩾0.001 level.

MEQ, Morningness–Eveningness Questionnaire; PSQI, Pittsburgh Sleep Quality Index; SD, standard deviation.

## Discussion

This is one of the first studies to evaluate sleep outcomes using validated, sleep-specific PROMs (PSQI and MEQ), in patients with TRD treated with IV ketamine. Our results support preliminary reports that used sleep subitems from standardized depression scales, that IV ketamine treatment for depression is associated with a perceived improvement in sleep. In our study, mood improved immediately, whereas total sleep quality improved later. The time difference from T0 (baseline) to T1 (before second infusion) would have been only 2–5 days (as per the hospital’s protocol of ketamine infusions occurring on Tuesdays and Thursdays), and lack of early improvement in sleep quality may reflect limits in reporting. Patients may need more consistent changes in sleep over a longer duration to report improvements. While it may be true that sleep improvement was secondary to mood improvement, one previous study found that patient-reported sleep improvement was a partial mediator of ketamine response and remission in depressed patients.^30^

Tendency toward ‘eveningness’ in the sleep phase has been previously related to depressive symptoms.^19^ In this study, both mood improvement and sleep phase advancement were noted with ketamine treatments, with patients moving toward less ‘eveningness’. This suggests that ketamine may have a chronotherapeutic effect, which is in keeping with recent theories of ketamine’s effects on the timekeeping of the central clock and alteration of the synchronization to external light cycles.^51^ It has been suggested that circadian changes represent a long-term downstream stabilization effect in mood disorders.^29^ A larger sample population and a further period of study beyond the endpoint used (1 week after the eighth treatment) may be needed to observe further chronotype changes. Nonetheless, our observations support a potential role for ketamine as a chronotherapeutic agent with normalization of circadian rhythm seen in its overall clinical effects.

Differences noted among patients with mixed features and those without are also of note. Patients with mixed features demonstrated improved sleep quality, while those without mixed features did not. Circadian rhythm advanced only in those without mixed features. Although we excluded individuals with bipolar depression, it remains possible that sleep in individuals with mixed features may be more similar to the sleep of individuals with bipolar depression. Moreover, patients with bipolarity display an instability in circadian rhythm. Circadian rhythm may be more disturbed and unstable in patients with bipolar depression,^52^ and it has been suggested that the clock genes underlying circadian cycles may differ in bipolar *versus* unipolar depression.^53^ This could account for the lack of circadian rhythm advancement among the individuals with mixed features in our study. It has been previously suggested that factors underlying TRD may include underlying bipolarity or mixed features. Ketamine shares several membrane stabilizing properties with other mood stabilizers, and has also demonstrated efficacy in bipolar depression in several randomized controlled trials (RCTs).^54–57^ While RCT data for esketamine’s efficacy in bipolar depression are limited, real-world use suggests a similar efficacy, and it has been suggested that ketamine and esketamine are promising mood stabilizers for individuals with bipolar disorder or depression with mixed features.^57^ This is a future area of research that could significantly advance the treatment of these patients.

The potential role of sleep in ketamine’s antidepressant effect has been previously reported.^25^ Slow-wave sleep (SWS) is reduced in depression, with improvements in SWS suggested as a potential mechanism of ketamine’s antidepressant action. An increase in SWS corresponds with a rapid increase in circulating BDNF, which is thought to elicit an increase in overall synaptic strength of cortical circuitry. This not only leads to improvements in depression, but also downstream effects potentially leading to improved sleep quality, and sleep homeostasis that interact with the circadian rhythm system.^58^ In this study, patients with mixed features did display an improvement in subjective sleep, while those without mixed features did not – this difference may be consistent with the concept that mixed features may share more in common with the sleep phenotype of bipolar depression, and that ketamine’s effects on sleep may be different in these populations. The significant differences between the mixed and nonmixed features groups, even in this small study, suggest a need for future studies looking at circadian effects of ketamine or other antidepressant treatments and to also identify and separately report these groups. Failure to do so may underestimate effects.

## Limitations

While the use of validated, sleep-specific PROMs is a strength of this study, it remains limited by the lack of objective data. Future studies could include objective measures such as wrist actigraphy. The study is also limited in its ability to evaluate the relationship between the improvements in mood and sleep. While the efficacy of IV ketamine for depression is well established, missing data precluded the ability to determine whether sleep was improving along with or independently of mood. Larger data sets with more complete mood data would be helpful to assess any differences in ketamine responders, remitters, and nonresponders. Due to the small sample size, and missing data, mood and sleep changes were reported as a whole group, which gives an idea of trend, and can serve as a basis for future data to further elucidate the relationship between mood, sleep, and ketamine. As there were such significant differences among the participants with mixed features *versus* those without, attempting to look at data in groups of responder/remitters *versus* nonresponders to ketamine would be confounded by the fact that some patients had mixed features. While the group of 29 did allow for statistically significant findings, it was too small to attempt subanalysis of 4 subgroups taking into account both of the above dimensions. Future, larger studies could report on responder/remitters *versus* nonresponders in each of the mixed features and no mixed features groups.

Despite these limitations, mood improvements noted in this study were consistent in keeping with previous systematic reviews, meta-analyses, and RCTs on clinical efficacy,^59^ and aligned with results from a recent systematic review and meta-analysis on real-world effectiveness trials.^60^ Also, repeated dosing showed mood continued to improve throughout the course of eight infusions, in keeping with current evidence that repeated dose ketamine may have a cumulative effect.^61^

Also, patients were followed naturalistically and could continue or change other medications and/or psychotherapy regimens according to recommendations from their treating psychiatrist. Hence, potential confounding effects due to natural changes or other treatments, including changes in antidepressants and psychotherapy, cannot be excluded. Similarly, dosing of the ketamine itself may have varied from 0.5 to 1 mg/kg, at the discretion of the treating psychiatrist. Despite these potential confounds, our study presents novel data to consider in the design of future studies to further elucidate the effects of ketamine on sleep.

## Conclusion

This observational study adds to growing evidence that repeated dose IV ketamine for depression is associated with improved sleep quality and advancement of circadian rhythm, while continuing to support previous evidence of its antidepressant effect in a real-world setting. Perhaps most interesting was our finding of differing effects on those with mixed features of depression *versus* those without. Future studies should take this into consideration and include larger populations to support subgroup analysis of depression, depression with mixed features, and bipolar depression, and evaluate responder/remitters *versus* nonresponders in each group. These patterns may further support theories into ketamine’s mechanism of action as an antidepressants and may eventually allow for tailoring of ketamine treatment to the subtype of depression. Future studies can more fully delineate the relationship between sleep and rapid improvement of depression with ketamine, and the chronotherapeutic effects of ketamine.

## Declarations

**Ethics approval and consent to participate:** This study was approved by the University of Alberta Health Research Ethics Board Pro00094438. Written informed consent was completed by all study participants.

**Consent for publication:** All participants signed written informed consent to participate in the study, which also indicated that anonymized data collected would be used for research dissemination, including potential publication.

**Availability of data and materials:** Not applicable.

## References

[1] Friedrich MJ. Depression is the leading cause of disability around the world. JAMA 2017; 317: 1517. 10.1001/jama.2017.3826 28418490

[2] Trivedi MH Rush AJ Wisniewski SR , et al. Evaluation of outcomes with citalopram for depression using measurement-based care in STAR*D: implications for clinical practice. Am J Psychiatry 2006; 163: 28–40. 16390886 10.1176/appi.ajp.163.1.28

[3] Gaynes BN Lux L Gartlehner G , et al. Defining treatment-resistant depression. Depress Anxiety 2020; 37: 134–145. 31638723 10.1002/da.22968

[4] Riemann D Krone LB Wulff K , et al. Sleep, insomnia, and depression. Neuropsychopharmacology 2020; 45: 74–89. 31071719 10.1038/s41386-019-0411-y PMC6879516

[5] Baglioni C Battagliese G Feige B , et al. Insomnia as a predictor of depression: a meta-analytic evaluation of longitudinal epidemiological studies. J Affect Disord 2011; 135: 10–19. 21300408 10.1016/j.jad.2011.01.011

[6] Pandi-Perumal SR Monti JM Burman D , et al. Clarifying the role of sleep in depression: a narrative review. Psychiatry Res 2020; 291: 113239. 32593854 10.1016/j.psychres.2020.113239

[7] Tsuno N Besset A Ritchie K. Sleep and depression. J Clin Psychiatry 2005; 66: 1254–1269. 16259539 10.4088/jcp.v66n1008

[8] Wichniak A Wierzbicka A Walęcka M , et al. Effects of antidepressants on sleep. Curr Psychiatry Rep 2017; 19: 63. 28791566 10.1007/s11920-017-0816-4 PMC5548844

[9] Franzen PL Buysse DJ. Sleep disturbances and depression: risk relationships for subsequent depression and therapeutic implications. Dialogues Clin Neurosci 2008; 10: 473–481. 19170404 10.31887/DCNS.2008.10.4/plfranzen PMC3108260

[10] Germain A Kupfer DJ. Circadian rhythm disturbances in depression. Hum Psychopharmacol 2008; 23: 571–585. 18680211 10.1002/hup.964 PMC2612129

[11] Moulton CD Pickup JC Ismail K. The link between depression and diabetes: the search for shared mechanisms. Lancet Diabetes Endocrinol 2015; 3: 461–471. 25995124 10.1016/S2213-8587(15)00134-5

[12] Cheng W Rolls ET Ruan H , et al. Functional connectivities in the brain that mediate the association between depressive problems and sleep quality. JAMA Psychiatry 2018; 75: 1052–1061. 30046833 10.1001/jamapsychiatry.2018.1941 PMC6233808

[13] Tononi G Cirelli C. Sleep and the price of plasticity: from synaptic and cellular homeostasis to memory consolidation and integration. Neuron 2014; 81: 12–34. 24411729 10.1016/j.neuron.2013.12.025 PMC3921176

[14] Alitalo O Saarreharju R Henter ID , et al. A wake-up call: sleep physiology and related translational discrepancies in studies of rapid-acting antidepressants. Prog Neurobiol 2021; 206: 102140. 34403718 10.1016/j.pneurobio.2021.102140 PMC9583188

[15] Zaki NFW Spence DW BaHammam AS , et al. Chronobiological theories of mood disorder. Eur Arch Psychiatry Clin Neurosci 2018; 268: 107–118. 28894915 10.1007/s00406-017-0835-5

[16] Gebara MA Siripong N DiNapoli EA , et al. Effect of insomnia treatments on depression: a systematic review and meta-analysis. Depress Anxiety 2018; 35: 717–731. 29782076 10.1002/da.22776

[17] Dodd S Bauer M Carvalho AF , et al. A clinical approach to treatment resistance in depressed patients: what to do when the usual treatments don’t work well enough? World J Biol Psychiatry 2021; 22: 483–494. 33289425 10.1080/15622975.2020.1851052

[18] Geoffroy PA Palagini L. Biological rhythms and chronotherapeutics in depression. Prog Neuropsychopharmacol Biol Psychiatry 2021; 106: 110158. 33152388 10.1016/j.pnpbp.2020.110158

[19] Au J Reece J. The relationship between chronotype and depressive symptoms: a meta-analysis. J Affect Disord 2017; 218: 93–104. 28463712 10.1016/j.jad.2017.04.021

[20] Asarnow LD Soehner AM Harvey AG. Circadian rhythms and psychiatric illness. Curr Opin Psychiatry 2013; 26: 566–571. 24060916 10.1097/YCO.0b013e328365a2fa PMC4000560

[21] Quera-Salva M-A Lemoine P Guilleminault C. Impact of the novel antidepressant agomelatine on disturbed sleep–wake cycles in depressed patients. Hum Psychopharmacol 2010; 25: 222–229. 20373473 10.1002/hup.1112

[22] Abdallah CG Adams TG Kelmendi B , et al. Ketamine’s mechanism of action: a path to rapid-acting antidepressants. Depress Anxiety 2016; 33: 689–697. 27062302 10.1002/da.22501 PMC4961540

[23] Swainson J McGirr A Blier P , et al. The Canadian Network for Mood and Anxiety Treatments (CANMAT) task force recommendations for the use of racemic ketamine in adults with major depressive disorder: recommandations du groupe de travail du réseau canadien pour les traitements de l’humeur et de l’anxiété (canmat) concernant l’utilisation de la kétamine racémique chez les adultes souffrant de trouble dépressif majeur. Can J Psychiatry 2021; 66: 113–125. 33174760 10.1177/0706743720970860 PMC7918868

[24] Liu B Liu J Wang M , et al. From serotonin to neuroplasticity: evolvement of theories for major depressive disorder. Front Cell Neurosci 2017; 11: 305. 29033793 10.3389/fncel.2017.00305 PMC5624993

[25] Matveychuk D Thomas RK Swainson J , et al. Ketamine as an antidepressant: overview of its mechanisms of action and potential predictive biomarkers. Ther Adv Psychopharmacol 2020; 10: 2045125320916657. 10.1177/2045125320916657 PMC7225830 32440333

[26] Duncan WC Slonena E Hejazi NS , et al. Motor-activity markers of circadian timekeeping are related to ketamine’s rapid antidepressant properties. Biol Psychiatry 2017; 82: 361–369. 28457485 10.1016/j.biopsych.2017.03.011 PMC5546993

[27] Bellet MM Vawter MP Bunney BG , et al. Ketamine influences CLOCK:BMAL1 function leading to altered circadian gene expression. PLoS One 2011; 6: e23982. 10.1371/journal.pone.0023982 PMC3161090 21887357

[28] Song B Zhu J-C. Mechanisms of the rapid effects of ketamine on depression and sleep disturbances: a narrative review. Front Pharmacol 2021; 12: 782457. 34970147 10.3389/fphar.2021.782457 PMC8712478

[29] Kohtala S Alitalo O Rosenholm M , et al. Time is of the essence: coupling sleep–wake and circadian neurobiology to the antidepressant effects of ketamine. Pharmacol Ther 2021; 221: 107741. 33189715 10.1016/j.pharmthera.2020.107741

[30] Rodrigues NB McIntyre RS Lipsitz O , et al. Do sleep changes mediate the anti-depressive and anti-suicidal response of intravenous ketamine in treatment-resistant depression? J Sleep Res 2022; 31: e13400. 10.1111/jsr.13400 34137095

[31] Wang M Zhang B Zhou Y , et al. Sleep improvement is associated with the antidepressant efficacy of repeated-dose ketamine and serum BDNF levels: a *post-hoc* analysis. Pharmacol Rep 2021; 73: 594–603. 33387333 10.1007/s43440-020-00203-1

[32] Pennybaker SJ Niciu MJ Luckenbaugh DA , et al. Symptomatology and predictors of antidepressant efficacy in extended responders to a single ketamine infusion. J Affect Disord 2017; 208: 560–566. 27839782 10.1016/j.jad.2016.10.026 PMC5154889

[33] Stultz DJ Stanley N Gills T , et al. Three months of treatment with esketamine: effects on depression, insomnia, and weight. Prim Care Companion CNS Disord 2020; 22: 19l02555. 10.4088/PCC.19l02555 32649066

[34] Vanderschelden B Gebara MA Oughli HA , et al. Change in patient-centered outcomes of psychological well-being, sleep, and suicidality following treatment with intravenous ketamine for late-life treatment-resistant depression. Int J Geriatr Psychiatry 2023; 38: e5964. 10.1002/gps.5964 37392089

[35] Zhuo C Tian H Li G , et al. Effects of ketamine on circadian rhythm and synaptic homeostasis in patients with treatment-resistant depression: a protocol for mechanistic studies of its rapid and sustained antidepressant actions in humans. Brain Behav 2019; 9: e01423. 10.1002/brb3.1423 PMC6851815 31617335

[36] Duncan WC Zarate CA. Ketamine, sleep, and depression: current status and new questions. Curr Psychiatry Rep 2013; 15: 394. 23949569 10.1007/s11920-013-0394-z PMC3827949

[37] von Elm E Altman DG Egger M , et al. Strengthening the Reporting of Observational Studies in Epidemiology (STROBE) statement: guidelines for reporting observational studies. BMJ 2007; 335: 806–808. 17947786 10.1136/bmj.39335.541782.AD PMC2034723

[38] Buysse DJ Reynolds CF Monk TH , et al. The Pittsburgh Sleep Quality Index: a new instrument for psychiatric practice and research. Psychiatry Res 1989; 28: 193–213. 2748771 10.1016/0165-1781(89)90047-4

[39] Huang Y Zhu M. Increased Global PSQI Score is associated with depressive symptoms in an adult population from the United States. Nat Sci Sleep 2020; 12: 487–495. 32765145 10.2147/NSS.S256625 PMC7381800

[40] Horne JA Ostberg O. A self-assessment questionnaire to determine morningness–eveningness in human circadian rhythms. Int J Chronobiol 1976; 4: 97–110. 1027738

[41] Iwasaki M Hirose T Mita T , et al. Morningness–eveningness questionnaire score correlates with glycated hemoglobin in middle-aged male workers with type 2 diabetes mellitus. J Diabetes Investig 2013; 4: 376–381. 10.1111/jdi.12047 PMC4020233 24843683

[42] Silva ACPE Dos Santos MJ Góes Gitaí DL , et al. Depression and anxiety symptoms correlate with diurnal preference, sleep habits, and Per3 VNTR polymorphism (rs57875989) in a non-clinical sample. J Affect Disord 2020; 277: 260–270. 32841827 10.1016/j.jad.2020.07.138

[43] Lam RW McIntosh D Wang J , et al. Canadian Network for Mood and Anxiety Treatments (CANMAT) 2016 clinical guidelines for the management of adults with major depressive disorder: section 1. Disease burden and principles of care. Can J Psychiatry 2016; 61: 510–523. 27486151 10.1177/0706743716659416 PMC4994789

[44] Zimmerman M Walsh E Friedman M , et al. Identifying remission from depression on 3 self-report scales. J Clin Psychiatry 2017; 78: 177–183. 28234434 10.4088/JCP.16m10641

[45] Zimmerman M D’Avanzato C Attiullah N , et al. Scoring rules and rating formats of Self-report Depression Questionnaires: a comparison of approaches. Psychiatry Res 2014; 218: 225–228. 24745466 10.1016/j.psychres.2014.03.025

[46] Zimmerman M Chelminski I McGlinchey JB , et al. A clinically useful depression outcome scale. Compr Psychiatry 2008; 49: 131–140. 18243884 10.1016/j.comppsych.2007.10.006

[47] Zimmerman M Martinez J Attiullah N , et al. Determining remission from depression on two self-report symptom scales: a comparison of the Quick Inventory of Depressive Symptomatology and the Clinically Useful Depression Outcome Scale. Compr Psychiatry 2012; 53: 1034–1038. 22520091 10.1016/j.comppsych.2012.03.001

[48] Stahl SM Morrissette DA Faedda G , et al. Guidelines for the recognition and management of mixed depression. CNS Spectr 2017; 22: 203–219. 28421980 10.1017/S1092852917000165

[49] Zimmerman M Chelminski I Young D , et al. A clinically useful self-report measure of the DSM-5 mixed features specifier of major depressive disorder. J Affect Disord 2014; 168: 357–362. 25103631 10.1016/j.jad.2014.07.021

[50] Fei Y Liu L Zheng D , et al. Reliability and validity of the Chinese version of the CUDOS-M in patients with mood disorders: a multicenter study across China. J Affect Disord 2021; 294: 723–729. 34343931 10.1016/j.jad.2021.07.014

[51] Sato S Bunney B Mendoza-Viveros L , et al. Rapid-acting antidepressants and the circadian clock. Neuropsychopharmacology 2022; 47: 805–816. 34837078 10.1038/s41386-021-01241-w PMC8626287

[52] Esaki Y Obayashi K Saeki K , et al. Association between circadian activity rhythms and mood episode relapse in bipolar disorder: a 12-month prospective cohort study. Transl Psychiatry 2021; 11: 525. 34645802 10.1038/s41398-021-01652-9 PMC8514471

[53] Lee H-J Son G-H Geum D. Circadian rhythm hypotheses of mixed features, antidepressant treatment resistance, and manic switching in bipolar disorder. Psychiatry Investig 2013; 10: 225–232. 10.4306/pi.2013.10.3.225 PMC3843013 24302944

[54] Yatham LN Kennedy SH Parikh SV , et al. Canadian Network for Mood and Anxiety Treatments (CANMAT) and International Society for Bipolar Disorders (ISBD) 2018 guidelines for the management of patients with bipolar disorder. Bipolar Disord 2018; 20: 97–170. 29536616 10.1111/bdi.12609 PMC5947163

[55] Bahji A Ermacora D Stephenson C , et al. Comparative efficacy and tolerability of adjunctive pharmacotherapies for acute bipolar depression: a systematic review and network meta-analysis. Can J Psychiatry 2021; 66: 274–288. 33174452 10.1177/0706743720970857 PMC7958200

[56] d’Andrea G Pettorruso M Lorenzo GD , et al. Rethinking ketamine and esketamine action: are they antidepressants with mood-stabilizing properties? Eur Neuropsychopharmacol 2023; 70: 49–55. 36867895 10.1016/j.euroneuro.2023.02.010

[57] Dell’Osso B Martinotti G. Exploring the potential of esketamine in the treatment of bipolar depression. Eur Neuropsychopharmacol 2023; 77: 21–23. 37660440 10.1016/j.euroneuro.2023.08.498

[58] Duncan WC Ballard ED Zarate CA. Ketamine-induced glutamatergic mechanisms of sleep and wakefulness: insights for developing novel treatments for disturbed sleep and mood. Handb Exp Pharmacol 2019; 253: 337–358. 28939975 10.1007/164_2017_51 PMC5866161

[59] McIntyre RS Rosenblat JD Nemeroff CB , et al. Synthesizing the evidence for ketamine and esketamine in treatment-resistant depression: an international expert opinion on the available evidence and implementation. Am J Psychiatry 2021; 178: 383–399. 33726522 10.1176/appi.ajp.2020.20081251 PMC9635017

[60] Alnefeesi Y Chen-Li D Krane E , et al. Real-world effectiveness of ketamine in treatment-resistant depression: a systematic review & meta-analysis. J Psychiatr Res 2022; 151: 693–709. 35688035 10.1016/j.jpsychires.2022.04.037

[61] Phillips JL Norris S Talbot J , et al. Single, repeated, and maintenance ketamine infusions for treatment-resistant depression: a randomized controlled trial. Am J Psychiatry 2019; 176: 401–409. 30922101 10.1176/appi.ajp.2018.18070834

